# Association between stroke and psychosis across four nationally representative psychiatric epidemiological studies

**DOI:** 10.1192/bjo.2023.47

**Published:** 2023-04-17

**Authors:** Vaughan Bell, William Tamayo-Agudelo, Grace Revill, David Okai, Norman Poole

**Affiliations:** Research Department of Clinical, Educational and Health Psychology, University College London, UK; and Department of Neuropsychiatry, South London and Maudsley NHS Foundation Trust, London, UK; Research Department of Clinical, Educational and Health Psychology, University College London, UK; and Universidad Cooperativa de Colombia, Medellín, Colombia; Research Department of Clinical, Educational and Health Psychology, University College London, UK; Department of Neuropsychiatry, South London and Maudsley NHS Foundation Trust, London, UK; Department of Neuropsychiatry, South West London and St George's Mental Health NHS Trust, London, UK

**Keywords:** Stroke, psychotic, delusions, hallucinations, neuropsychiatry

## Abstract

**Background:**

Both stroke and psychosis are independently associated with high levels of disability. However, psychosis in the context of stroke has been under-researched. To date, there are no general population studies on their joint prevalence and association.

**Aims:**

To estimate the joint prevalence of stroke and psychosis and their statistical association using nationally representative psychiatric epidemiology studies from two high-income countries (the UK and the USA) and two middle-income countries (Chile and Colombia) and, subsequently, in a combined-countries data-set.

**Method:**

Prevalences were calculated with 95% confidence intervals. Statistical associations between stroke and psychosis and between stroke and psychotic symptoms were tested using regression models. Overall estimates were calculated using an individual participant level meta-analysis on the combined-countries data-set. The analysis is available online as a computational notebook.

**Results:**

The overall prevalence of probable psychosis in stroke was 3.81% (95% CI 2.34–5.82) and that of stroke in probable psychosis was 3.15% (95% CI 1.94–4.83). The odds ratio of the adjusted association between stroke and probable psychosis was 3.32 (95% CI 2.05–5.38). On the individual symptom level, paranoia, hallucinated voices and thought passivity delusion were associated with stroke in the unadjusted and adjusted analyses.

**Conclusions:**

Rates of association between psychosis and stroke suggest there is likely to be a high clinical need group who are under-researched and may be poorly served by existing services.

Stroke and active psychosis are independently considered to cause some of the highest levels of disability among all health conditions, meaning their combination is likely to result in severely debilitating outcomes for affected individuals. To add to the complexity, initial evidence suggests that the most common treatment for psychosis in stroke-affected individuals (antipsychotics) may increase mortality in this patient group.^1^

Despite the clear need for better evidence to inform care for affected patients, the first systematic review – and indeed the first review – dedicated to stroke and psychosis was only published in 2018 and focused specifically on post-stroke psychosis.^2^ However, it is important to note that the association between stroke and psychosis extends beyond cases of post-stroke psychosis and also includes those who have a preceding history of psychosis and are later affected by stroke. Indeed, people with a diagnosis of schizophrenia are at an increased risk of stroke and recurrent stroke.^3^ The main treatment for psychosis, antipsychotic medication, raises the risk of stroke with evidence for a causal role of metabolic syndrome and cardiac arrhythmias.^4^ Studies on predictors of cardiovascular events more generally in patients with schizophrenia also highlight the role of shared risk factors that may raise the risk of both conditions independently.^5^

One difficulty in estimating the level of association between stroke and psychosis is that as far as we are aware, all existing estimates have been drawn from clinical studies rather than population studies, meaning it is not clear to what extent estimates might be affected by selection biases, particularly referral bias. For example, all studies used to estimate prevalence of delusions and hallucinations in stroke patients included in the meta-analysis reported by Stangeland et al^2^ were drawn from hospital-admitted stroke patients, potentially oversampling patients with the highest levels of disability. Indeed, hospital admission referral biases have been evidenced for both stroke^6^ and psychosis.^7^

These limitations are particularly important when trying to estimate the association between stroke and psychosis in low- and middle-income countries, where specialised stroke care may be less available. Indeed, low- and middle-income countries proportionally have the highest levels of stroke incidence and poor outcomes,^8,9^ raising the possibility of whether stroke and psychosis might be a more frequent combination in such countries.

Consequently, in this study, we identified epidemiological studies that recorded both stroke and psychosis from four countries and aimed to estimate the joint prevalence of stroke and psychosis and their statistical association. These included two high-income countries (the UK and the USA) and two middle-income countries (Chile and Colombia). Each of these countries have completed nationally representative psychiatric epidemiology studies that included structured assessments of psychotic disorders and/or psychotic symptoms as well as measures of the participants’ health, including stroke status. We subsequently combined all national data-sets into a single data-set to conduct an individual participant-level meta-analysis to estimate the overall prevalence and association between stroke and psychosis across all four countries.

## Method

### Data-sets

We used four nationally representative psychiatric epidemiological studies that recorded presence of both stroke and psychosis from the UK, the USA, Chile and Colombia. Matched variables from across the four data-sets were also merged to create a single combined-countries data-set, which we used to conduct an internal individual participant-level meta-analysis. The original data-sets are described below.

### Adult Psychiatric Morbidity Survey 2007 (England, UK)

The Adult Psychiatric Morbidity Survey 2007 was a household survey that used multi-stage stratified probability sampling to recruit participants. Using the English national postcode database, private households were identified, and any resident individual aged 16 years or over was invited to participate. If more than one individual aged over 16 years was resident, one adult was randomly chosen to ensure the same chance of being selected for all eligible individuals. Psychotic symptoms were measured using the Psychosis Screening Questionnaire,^10^ a 20-item interview that measures the presence of symptoms of hypomania, thought interference, persecution, perceptual abnormalities, strange experiences and hallucinosis. Full details of the survey, sampling methods and consent procedure have been reported by McManus et al.^11^

### Collaborative Psychiatric Epidemiology Surveys 2001–2003 (USA)

The Collaborative Psychiatric Epidemiology Surveys consisted of the three nationally representative surveys of mental health in the USA: the National Comorbidity Survey Replication, the National Study of American Life, and the National Latino and Asian American Study of Mental Health. A two-component sampling method was used to recruit participants. The first component involved a multistage stratified area probability design to derive a nationally representative household sample, and the second involved high-density supplemental sampling to oversample specific ethnic groups (African–Caribbean, Chinese, Filipino, Vietnamese and Puerto Rican). Psychotic symptoms were measured using the World Health Organization Composite International Diagnostic Interview (WHO-CIDI) 3.0 Psychosis Screen,^12^ which measures the lifetime presence of six symptoms: visual hallucinations, auditory hallucinations, thought insertion, thought control, delusions of reference and persecutory delusions. Full details of the survey, sampling methods and consent procedure are given by Heeringa et al.^13^

### National Mental Health Survey 2015 (Colombia)

The National Mental Health Survey 2015 (Encuesta Nacional de Salud Mental) was a national survey in Colombia completed by the Ministry of Health and Social Protection (Ministerio de Salud y Protección Social). Participants were recruited using multistage stratified sampling that involved stratifying the population by region, municipality and geographical area. Neighbourhood blocks in urban areas and municipalities in rural areas were selected, and all households were contacted for participation. Psychotic symptoms were measured using the WHO Self Reporting Questionnaire 24 (SRQ-24),^14^ which was deployed as an interview rather than a self-completion questionnaire. The SRQ-24 measures the presence of four psychotic symptoms: persecutory delusion, grandiosity, thought interference and auditory hallucinations. Full details of the survey, sampling methods and consent procedure have been reported by Gómez-Restrepo et al.^15^

### National Health Survey 2016–2017 (Chile)

The National Health Survey 2016–2017 (Encuesta Nacional de Salud) was a national survey by the Chilean Ministry of Health of non-institutionalised individuals aged 15 years and older in households in urban and rural areas across 15 regions of Chile. Participants were identified using stratified multistage probability sampling. Psychotic symptoms were measured with the WHO-CIDI 3.0 Psychosis Screen.^12^ Full details of the survey, sampling methods and consent procedure are given by the Ministerio de Salud.^16^

### Ethics

The authors assert that all procedures contributing to this work comply with the ethical standards of the relevant national and institutional committees on human experimentation and with the Helsinki Declaration of 1975, as revised in 2008. This study is a secondary data analysis of data-sets that exist in the public domain, and ethics approval for this human study was waived by the University College London Research Ethics Committee. As shown in Table 1, different countries’ studies had different lower ages for their definition of adults (from 16–18 years), but all participants consented as adults and provided written informed consent for participation in the original studies.

**Table 1:** Descriptive statistics for national and combined data-sets

	UK (*N* = 7403)	USA (*N* = 6082)	Colombia (*N* = 10870)	Chile (*N* = 3403)	Combined (*N* = 27 758)
Age, years					
Mean (s.d.)	51.1 (18.6)	41.5 (15.8)	43.4 (16.8)	49.4 (17.9)	45.8 (17.7)
Median	50.0	39.0	42.0	50.0	44.0
[min, max]	[16.0, 95.0]	[18.0, 99.0]	[18.0, 96.0]	[17.0, 98.0]	[16.0, 99.0]
Sex					
Male	3197 (43.2%)	2747 (45.2%)	4384 (40.3%)	1226 (36.0%)	11554 (41.6%)
Female	4206 (56.8%)	3335 (54.8%)	6486 (59.7%)	2177 (64.0%)	16204 (58.4%)
Highest level of education					
No/primary education	2278 (30.8%)	540 (8.9%)	4007 (36.9%)	766 (22.5%)	7591 (27.3%)
Mid-teen high school	2103 (28.4%)	835 (13.7%)	5152 (47.4%)	895 (26.3%)	8985 (32.4%)
Late-teen high school	938 (12.7%)	3604 (59.3%)	895 (8.2%)	1040 (30.6%)	6477 (23.3%)
College/university	1916 (25.9%)	1103 (18.1%)	713 (6.6%)	668 (19.6%)	4400 (15.9%)
Missing	168 (2.3%)	0 (0%)	103 (0.9%)	34 (1.0%)	305 (1.1%)
Stroke					
Yes	180 (2.4%)	171 (2.8%)	79 (0.7%)	95 (2.8%)	525 (1.9%)
No	7223 (97.6%)	5724 (94.1%)	10791 (99.3%)	3287 (96.6%)	27025 (97.4%)
Missing	0 (0%)	187 (3.1%)	0 (0%)	21 (0.6%)	208 (0.7%)
Paranoia					
Yes	569 (7.7%)	68 (1.1%)	2340 (21.5%)	59 (1.7%)	3036 (10.9%)
No	6834 (92.3%)	4926 (81.0%)	8530 (78.5%)	3304 (97.1%)	23594 (85.0%)
Missing	0 (0%)	1088 (17.9%)	0 (0%)	40 (1.2%)	1128 (4.1%)
Hallucinated voices					
Yes	68 (0.9%)	293 (4.8%)	362 (3.3%)	152 (4.5%)	875 (3.2%)
No	7335 (99.1%)	4702 (77.3%)	10508 (96.7%)	3211 (94.4%)	25756 (92.8%)
Missing	0 (0%)	1087 (17.9%)	0 (0%)	40 (1.2%)	1127 (4.1%)
Passivity delusion					
Yes	77 (1.0%)	33 (0.5%)	534 (4.9%)	18 (0.5%)	662 (2.4%)
No	7326 (99.0%)	4962 (81.6%)	10336 (95.1%)	3345 (98.3%)	25969 (93.6%)
Missing	0 (0%)	1087 (17.9%)	0 (0%)	40 (1.2%)	1127 (4.1%)
Probable psychosis					
Yes	66 (0.9%)	36 (0.6%)	505 (4.6%)	27 (0.8%)	634 (2.3%)
No	7337 (99.1%)	6046 (99.4%)	10365 (95.4%)	3376 (99.2%)	27124 (97.7%)

### Variable coding and missing data management

Symptom data were recoded to represent strong evidence for the presence of symptoms. Where there was an ambiguous response data in response to interview questions about psychotic symptoms (‘unsure’ responses in the UK Adult Psychiatric Morbidity Survey; ‘don't know’ or ‘didn't respond’ responses in the Chile National Health Survey), these were recoded as absent. Ambiguous responses were present at low rates: 0.4% of responses in the UK Adult Psychiatric Morbidity Survey data and 0.1% of responses in the Chile National Health Survey data.

In the UK Adult Psychiatric Morbidity Survey, psychosis and stroke data was only collected after the participant responded ‘yes’ to initial screening questions, meaning missing data represented questions being intentionally not asked and therefore data was not missing at random. Consequently, the presence of stroke or psychotic symptoms was coded as not present for ‘no’ or ‘item not applicable’ data.

Missing data as originally present in the data-set are reported in Table 1. Notably, the majority of variables with missing data had low levels of missingness, below the threshold of 5% considered to be likely to bias estimates.^17^ However, the psychotic symptom data from the US Collaborative Psychiatric Epidemiology Surveys data showed higher levels of missingness (approximately 18%). Random forest missing data imputation is highly reliable in reducing bias in estimates.^18^ Consequently, the missing psychotic symptoms values were imputed using the missForest package for R. No additional instances of symptoms were imputed for the Collaborative Psychiatric Epidemiology Surveys data and so all missing variables were coded as ‘not present’.

### Variables

#### Exposure

In all studies, individuals were asked to report whether they had been diagnosed with stroke by a doctor during the structured health assessment. This was used as the primary exposure variable in the regression analyses.

#### Outcome

Owing to the use of differing psychosis measures across studies, we extracted symptom-level items from interviews that measured the presence of the following symptoms across all four studies: (a) paranoia, (b) hallucinated voices and (c) thought passivity delusion.

There was no consistent metric for probable psychosis across surveys. The Adult Psychiatric Morbidity Survey and the Collaborative Psychiatric Epidemiology Surveys had inconsistent criteria (the former was based solely on symptom screening, the latter included service use – i.e. antipsychotics, hospital admission), and the other surveys did not code for this category. Therefore, we created standardised criteria for the category of ‘probable psychosis’, which was coded when any two psychotic symptoms were present. Consequently, probable psychosis was coded when a participant reported at least one delusion-like belief along with hallucinated voices, or at least two delusion-like beliefs, at least one of which was a thought-passivity experience.

#### Potential confounders

A graph analysis that mapped major evidenced risk factors between stroke and psychosis^19^ indicated that the total effect between stroke and psychosis could not be estimated by covariate adjustment, largely owing to the reciprocal causal relationship between stroke and psychosis and the role of alcohol and smoking, which act as mediators.

However, we selected a minimal group of potential confounders that were most likely to represent pre-onset risk factors for both stroke and psychosis, namely age, sex and highest level of education, to help refine the estimate. Age and sex are independent predictors of stroke^20^ and psychosis^21^ before onset. There was no consistent measure of pre-onset socioeconomic status in any of the four studies. However, highest level of education, which is correlated strongly with socioeconomic status and is frequently used as a component measure of it,^22^ is a pre-onset predictor of both stroke^23^ and psychosis^24^ and was included. Highest educational attainment was recoded across studies to a consistent coding of ‘no or primary education only’, ‘mid-teen high school’, ‘late teen high school’, and ‘college/university’.

Both alcohol use and smoking are likely to be independent risk factors for both stroke^25,26^ and psychosis.^27,28^ However, there is also strong evidence for psychosis as a causal risk factor for smoking and alcohol use,^29^ potentially indicating its additional role as a mediating factor. Furthermore, smoking and alcohol intake were only measured contemporaneously in the studies that reported them. Given these issues, alcohol and smoking were not included as potential confounders in the analysis.

### Analysis

All analysis was conducted using R version 4.0.3, and the full code and output for the analysis is available in the format of a Jupyter Notebook, a document that combines both code and output in a form that can be re-run and reproduced. All analysis code is available at the following link: https://github.com/vaughanbell/stroke-psychosis-national-epi-analysis.

### Prevalence

We calculated the prevalence of stroke, prevalence of probable psychosis, prevalence of probable psychosis in people with stroke and prevalence of stroke in people with probable psychosis using the epiR package. Prevalences and 95% confidence intervals were calculated for each of these for each individual national study and meta-analytically using the combined-countries data-set.

### Association between stroke and psychosis

At the national data-set level, we used logistic regression models to estimate the associations between stroke, probable psychosis and individual psychotic symptoms (paranoia, hallucinated voices, passivity delusion). We first estimated the unadjusted association and then the adjusted association – adjusted for sex, age and highest level of education. Survey weights were of an incompatible format between data-sets and so were not included in the analysis.

### Individual participant-level meta-analysis

We completed an internal meta-analysis of individual participant data by additionally conducting prevalence and regression analyses on the combined-countries data-set. Following recommendations from Riley et al,^30^ when completing the regression analyses we accounted for potential clustering of participants within studies by using multi-level regression models where country was added as a random effect.

## Results

Descriptive statistics for each national survey and the combined data-set are reported in Table 1. The demographic profile was broadly similar across national surveys.

### Prevalence

Table 2 displays the calculated prevalence with 95% confidence intervals for stroke, probable psychosis, probable psychosis in stroke and stroke in probable psychosis. The larger estimates of prevalence within national surveys tend to be accompanied by wider confidence intervals, although the estimates for the combined-countries data-set had consistently narrower confidence intervals, suggesting more reliable estimates.

**Table 2:** Prevalence and 95% confidence intervals for stroke, probable psychosis, probable psychosis in stroke and stroke in probable psychosis across the four nations and combined-country data-sets

	Prevalence (95% CI)			
	Stroke in total population	Probable psychosis in total population	Probable psychosis in stroke population	Stroke in probable psychosis population
UK	2.43% (2.09–2.81)	0.89% (0.69–1.13)	1.11% (0.13–3.96)	3.03% (0.37–10.52)
USA	2.90% (2.49–3.36)	0.59% (0.41–0.82)	3.51% (1.30–7.48)	16.67% (6.37–32.81)
Colombia	0.73% (0.58–0.90)	4.65% (4.26–5.06)	13.92% (7.16–23.55)	2.18% (1.09–3.86)
Chile	2.81% (2.28–3.42)	0.79% (0.52–1.15)	1.05% (0.03–5.73)	3.70% (0.09–18.97)
Combined meta-analytic estimate	1.91% (1.75–2.07)	2.28% (2.11–2.47)	3.81% (2.34–5.82)	3.15% (1.94–4.83)

### Association and adjusted associations between stroke and psychosis

Unadjusted associations between stroke and psychosis alongside associations adjusted for potential confounders are reported in Table 3. In addition, Table 4 reports unadjusted and adjusted associations for specific symptoms of psychosis.

**Table 3:** Results of unadjusted and adjusted logistic regression analyses reporting associations between stroke and probable psychosis with 95% confidence intervals

	Odds ratio (95% CI)	
	Unadjusted	Adjusted
Probable psychosis		
UK	1.26 (0.31–5.17)	1.11 (0.15–8.26)
USA	6.90 (2.83–16.81)	6.22 (2.52–15.35)
Colombia	3.37 (1.77–6.42)	4.04 (2.10–7.78)
Chile	1.33 (0.18–9.94)	1.33 (0.18–10.09)
Combined meta-analytic estimate	3.06 (1.93–4.85)	3.32 (2.05–5.38)

**Table 4:** Results of unadjusted and adjusted logistic regression analyses reporting associations between stroke and psychotic symptoms with 95% confidence intervals

	Odds ratio (95% CI)	
	Unadjusted	Adjusted
*Paranoia*		
UK	0.63 (0.32–1.23)	0.99 (0.46–2.16)
USA	2.71 (1.07–6.82)	2.50 (0.99–6.34)
Colombia	2.01 (1.27–3.20)	2.34 (1.46–3.74)
Chile	1.92 (0.59–6.26)	1.71 (0.52–5.66)
Combined meta-analytic estimate	1.38 (1.00–1.90)	1.66 (1.19–2.32)
*Hallucinated voices*		
UK	0.60 (0.08–4.32)	0.85 (0.11–6.29)
USA	2.33 (1.41–3.86)	2.03 (1.22–3.38)
Colombia	4.30 (2.20–8.41)	4.06 (2.05–8.03)
Chile	0.71 (0.22–2.26)	0.71 (0.22–2.29)
Combined meta-analytic estimate	2.01 (1.39–2.90)	1.89 (1.30–2.74)
*Thought passivity delusion*		
UK	1.07 (0.26–4.40)	0.67 (0.09–4.94)
USA	2.17 (0.52–9.16)	2.12 (0.50–9.00)
Colombia	3.52 (1.89–6.55)	4.27 (2.27–8.03)
Chile	2.09 (0.28–15.87)	2.17 (0.28–16.95)
Combined meta-analytic estimate	2.51 (1.52–4.14)	2.68 (1.59–4.52)

In the regression analyses, stroke did not reliably predict probable psychosis in the UK or Chile, although it was a reliable predictor in the USA and Colombia and in the combined-countries data-set. There was variation in the extent to which stroke was reliably associated with individual psychotic symptom measures across countries, although stroke was reliably associated with all symptoms in the combined-countries data-set.

## Discussion

We report the joint prevalences of stroke and probable psychosis across four nationally representative epidemiological studies and subsequently the association between stroke and probable psychosis after adjustment for potential confounders. We subsequently calculated overall estimates from a combined-countries data-set using individual participant-level meta-analysis. We found that the prevalence of probable psychosis in people with stroke ranged from 1.05% (Chile) to 13.92% (Colombia), with the prevalence from the combined-countries data-set estimated at 3.81%. Conversely, the prevalence of stroke in people with probable psychosis ranged from 2.18% (Colombia) to 16.67% (US), with the combined-countries prevalence estimated at 3.15%. However, the larger estimates were accompanied by wide confidence intervals and are less likely to be accurate estimates of true population prevalence. Estimates for the adjusted association between stroke and probable psychosis ranged from an odds ratio of 1.11 (95% CI 0.15–8.26) in the UK to an odds ratio of 6.22 in the US (95% CI 2.52–15.35), with the association from the combined data-set estimated at 3.32 (95% CI 2.05–5.38). We also examined the association between stroke and paranoia, hallucinated voices and thought passivity delusion, and although we found significant variation in the reliability and strength of association across countries, all three psychotic symptoms were associated with stroke in the unadjusted and adjusted analyses in the combined-countries data-set.

This evidence suggests a relatively high co-prevalence of stroke and psychosis, with approximately 1 in 26 people with stroke having probable psychosis and 1 in 32 of people with probable psychosis having stroke across the combined-countries data-set. This is despite the fact that psychosis has often been described as a ‘rare’ complication of stroke in the literature and has mostly been reported as single case studies or case series. The estimate here is broadly in line with previous estimates of single psychotic symptoms in patients with stroke with meta-analytic estimates (admittedly from a small number of studies), suggesting a delusion prevalence of 4.67% and hallucination prevalence of 5.05%.^2^

We also note that the majority of research in this area has focused on post-stroke psychosis, which probably contributes only a proportion of the co-prevalence of stroke and psychosis. As psychosis and treatment for psychosis are risk factors for later stroke,^31,32^ stroke in patients with a preceding history of psychosis is also likely to be an important contributory factor to co-prevalence. We also note here that initial studies report that patients with psychosis who later experience stroke have worse outcomes and are less likely to receive equitable care,^33^ including timely invasive interventions.^4^ Taken together, this evidence suggests that stroke and psychosis may be highly disabling but is under-recognised and probably under-served by existing services.

It is important to note that significant international variability was found in the estimates of association – either through calculating prevalence or based on odds ratios – between stroke and psychosis. Given the variability of measures used to measure psychotic symptoms within countries, one key question is the extent to which these estimates are affected by characteristics of the measures versus the extent to which the prevalence of psychotic symptoms and their association with stroke varies between countries.

We note two standout prevalence figures: a prevalence of probable psychosis in stroke of 13.92% in Colombia and a prevalence of stroke in probable psychosis of 16.67% in the USA. Both of these figures had wide confidence intervals, and the accompanying alternative prevalences (stroke in probable psychosis in Colombia, and probable psychosis in stroke in the US) were within the more typical ranges internationally. This suggests that they may be less accurate estimates of the true prevalence. However, it remains challenging to separate measurement error from population-specific risk factors that contribute to these larger figures, given the cross-sectional nature of the data.

For example, the estimated rate of probable psychosis in the total population was markedly higher in Colombia, which also had the highest estimated rate of probable psychosis in stroke. We note here that several factors may have been important in influencing this outcome. The Colombia National Mental Health Survey used the WHO Self Reporting Questionnaire 24,^14^ which, although it was deployed in an interview format, solely relied on participant answers without any judgement from the trained interviewers regarding the likelihood of the answer representing a symptom. Although self-report questionnaires for psychotic symptoms show broad agreement with interview measures, they may overreport milder symptoms. We also note that of the four countries included in this analysis, Colombia has the highest rate, and an internationally high rate, of violence and victimisation as well as experience of a long-running armed conflict. This probably contributed both to the overrating of the paranoia item in terms of it measuring genuine threat rather than an exaggerated perception of threat, as well as probably increasing the rate of genuine paranoia as psychopathology, as violent victimisation is associated with a higher risk of subsequent psychosis.

The high-prevalence, wide-confidence-interval estimate of stroke in probable psychosis prevalence in the USA is likely to reflects the fact that the USA had the highest prevalence of stroke together with the lowest prevalence of psychosis reported here. There is some evidence that stroke prevalence may have been slightly overestimated in this study: the 2.9% US stroke prevalence reported here versus the 2.6% reported by the Centers for Disease Control and Prevention for non-institutionalised US adults in 2005.^34^ It is possible that psychosis prevalence was slightly underestimated. The Global Burden of Disease study reported higher rates of schizophrenia in the USA compared to the three other countries reported here,^35^ despite it having the lowest rate of psychosis estimated in this study.

One potential way to interpret this data is to compare the extent to which the prevalence of probable psychosis used in this study compares with the prevalence of psychosis phenotypes. Here, our probable psychosis category as applied to the UK, USA and Chile surveys is more likely to be measuring a narrow psychosis phenotype more akin to psychotic disorder, whereas in Colombia, it is more likely to be measuring a broader psychosis phenotype of psychotic experiences.^36^ However, it is also worth noting that in a recent systematic review of post-stroke psychosis, delusional disorder, typically involving a single isolated delusion, was the most commonly reported psychosis in post-stroke cases – albeit from a relatively poor-quality evidence base.^2^ The probable psychosis criteria used in this study would have excluded these cases, indicating that the full prevalence of psychosis may have been underestimated.

An additional factor is the extent to which stroke, psychosis and their possible combination may be underreported in community epidemiological studies owing to case ascertainment bias – in that those with more severe difficulties are less likely or less able to participate. Aked et al^37^ compared stroke ascertainment between a community epidemiological study and a clinical register and reported that the community study was more likely to detect milder strokes but equally likely to detect more severe cases. Nevertheless, the data used in the present study were from psychiatric epidemiology studies that required active participation in an extensive interview. It is likely that this may have led to an underrepresentation of more severe stroke or communication-impairing strokes in the data-set and, potentially, cases with more severe disability caused by a combination of stroke and psychosis.

We also note here that stroke was measured in all surveys by an interview item asking whether the person had been diagnosed with stroke by a doctor. Self-reported stroke has been found to have a consistently high negative predictive value but a variable positive predictive value (22–87%), with the misreporting of transient ischaemic attacks as stroke likely to be a major contributor to false positive reporting.^38^ However, the measure used in this study was not self-reported stroke *per se* but self-report of doctor-diagnosed stroke. As far as we are aware, the only study we know that has examined the accuracy of this specific method of reporting stroke was that of Walker et al,^39^ where self-report of doctor-diagnosed stroke had a positive predictive value of 0.89, with the majority of false positives being reports of transient ischaemic attacks. Hence, the measure included in this study is most likely to represent both stroke proper and transient ischaemic attack. In addition, this measure is likely to be affected by the number of doctors available to diagnose stroke. This may underestimate prevalence in lower-income countries where healthcare may be less accessible or inaccessible, or more likely to be carried out by non-physician healthcare professionals, particularly in remote or rural areas. The lack of detail beyond the presence or absence of stroke also means it was not possible to make inferences regarding the relationship between stroke type, severity, location, recurrence and psychosis. Accordingly, studies using formal diagnosis and additional data on stroke characteristics are needed to ensure the highest accuracy of estimates and associations.

We also note that the differing availability of mental health services could affect the prevalence of psychotic symptoms, because although each study measured psychotic symptoms by interviewing the participant directly, effective available treatment could reduce the presence of symptoms.

There are additional limitations that should be noted. Some potentially useful covariates could not be included because they were not measured in all data-sets. One is the extent to which the findings provide a guide to future stroke and psychosis prevalence, given the improving stroke survival rates in high-income countries, largely due to improvements in acute stroke care.^40^ Given the high rates of stroke risk factors in individuals with pre-existing psychosis, we suggest this will increase rates of post-psychosis stroke owing to better survival rates, and it is possible that this may increase the rates of post-stroke psychosis, although the relationship between stroke severity and psychosis risk is still poorly understood. We also note the increasing incidence of stroke in the young globally,^41^ potentially changing the risk profile of stroke and of comorbid stroke and psychosis.

As this study used cross-sectional data, the extent to which the association between stroke and psychosis consisted of post-stroke psychosis versus people with psychosis who later experienced stroke was impossible to determine. Longitudinal studies will be needed to address these key questions, and we note here that longitudinal studies examining to what extent psychosis occurs post-stroke and to what extent stroke occurs post-psychosis but, crucially, measured within the same cohort, are likely to be important in addressing these key issues. This information is clearly important in developing both preventative healthcare and understanding how specific services (specifically psychiatry and neurology) should prioritise treatment and referral, given that the order of which psychosis or stroke occurs is likely to determine which service a patient has first contact with.

We also suggest that involvement of more integrated psychological medicine services in stroke services including both psychiatry and psychology is likely to be important, as is prioritising management of stroke risk factors in patients with psychosis.^42^ In addition, psychiatrists should be aware of the signs and symptoms of stroke, including apparently ‘silent stroke’, and be aware of timely referral pathways to specialist stroke services in their area.

In conclusion, we report the first study on the association of stroke and psychosis in the general population that examines the co-prevalence and association within four countries: the USA, UK, Colombia and Chile. We note that the conditions co-occur more frequently than has previously been assumed and that there remains a marked lack of research in this area. This is a particular priority given the potentially high need of these patient groups and the potential avoidance of stroke if risk factors are appropriately managed. Future research needs to involve standardised diagnostic assessments and longitudinal studies to determine the extent to which stroke and psychosis appear in specific causal sequences.

## Data Availability

This study is a secondary analysis of epidemiological data that has been made available by the original research agencies for further research. All analysis code and links to the original data repositories are included in this study's online archive: https://github.com/vaughanbell/stroke-psychosis-national-epi-analysis.
